# Supplementary material for: The magnitude of anemia and associated factors among adult diabetic patients in Tertiary Teaching Hospital, Northern Ethiopia, 2019, cross-sectional study
Source: PLoS One. 2020 Nov 10;15(11):e0240678. doi: 10.1371/journal.pone.0240678 (PMC7671748; doi:10.1371/journal.pone.0240678)
Supplement: S1 Appendix — (ZIP) [file pone.0240678.s001.zip › S1 appendix.docx]

**English Version of the Questionnaire**

**Instruction:** circle the response from the alternatives

Part one: Socio-demographic characteristics

| **Age (years)** | 1. < 60 | **Religion** | 1. Orthodox |
| --- | --- | --- | --- |
|  | 1. > 60 |  | 1. Muslim |
| **Sex** | 1. Male |  | 1. Catholic |
|  | 1. Female |  | 1. Others (specify)___ |
| **Residence** | 1. Urban | **Ethnicity** | 1. Tigray |
|  | 1. Rural |  | 1. Afar |
| **Educational**  **status** | 1. No formal schooling |  | 1. Amhara |
|  | 1. Primary completed |  | 1. Others specify___ |
|  | 1. Secondary completed | **Occupational status** | 1. Government employee |
|  | 1. College/university |  | 1. Self employed |
|  | 1. Post graduate degree |  | 1. Student |
| **Marital status** | 1. Single |  | 1. Daily laborer |
|  | 1. Married |  | 1. Retired |
|  | 1. Separated |  | 1. Farmer |
|  | 1. Divorced |  | 1. Other specify_____ |
|  | 1. Widowed | **Level of income** | 1. High |
|  |  |  | 1. Medium |
|  |  |  | 1. Low |

**Part two: clinical status profile**

| **Patient profiles** | | **Responses** | **Patient profiles** | | **Responses** |  |
| --- | --- | --- | --- | --- | --- | --- |
| Type of diabetes | | 1. Type 1 | Was the patient on his/her regular Anti diabetic medications | | 1. Yes |  |
|  |  | 1. Type 2 |  |  | 1. No |  |
|  |  | 1. Others (specify_) | Duration with diabetes | | 1. <5 years |  |
| Type of treatment | | 1. Lente insulin |  |  | 1. 5-10 years |  |
|  |  | 1. Metformin |  |  | 1. >10 years |  |
| Does the patients has Any co-existing chronic disease | | 1. Yes | Does the patient has Retinopathy | | 1. Yes |  |
|  |  | 1. No |  |  | 1. No |  |
| Does the patient has Nephropathy | | 1. Yes |  |  | 1. Yes |  |
|  |  | 1. No | Does the patient have  Neuropathy | | 1. No |  |
| Smoking status | | 1. smoker |  |  | 1. Reactive |  |
|  |  | 1. Non-smoker | HIV/AIDS status | | 1. Non-reactive |  |
| Hypertension status | | 1. Yes |  |  | 1. Unknown |  |
|  |  | 1. No | Red blood cells  (million/ml) | | 1. <4.2 |  |
| eGFR(ml/min) | | 1. <60 |  |  | 1. 4. 2-5.4 |  |
|  |  | 1. ≥60 |  |  | 1. >5.4 | |
| Waist circumference | | 1. ≥88cm |  |  | 1. <4.7 | |
|  |  | 1. ≥102cm |  |  | 1. 4.7-6.1 | |
| Serum creatinine | | 1. <1.2 mg/dl |  |  | 1. >6.1 | |
|  |  | 1. >1.2mg/dl | Hematocrit  (In %) | female | 1. < 36 |  |
| Hemoglobin  (in mg/dl) | Female | 1. <12mg/dl |  |  | 1. >36 |  |
|  |  | 1. >12mg/dl |  | Male | 1. <39 |  |
|  | Male | 1. <13mg/dl |  |  | 1. >39 |  |
|  |  | 1. >13mg/dl | Weight of the patient (in kg) | | ________ |  |
| Systolic blood pressure | | _________mmHg | Height of the patient (in m) | | _______ |  |
| Diastolic blood pressure | | __________mmHg |  |  |  |  |
| Did you take any anti-helminthes drug in the last three months | | 1.Yes  2. No | Do you drink alcohol frequently? | | 1. Yes 2. No |  |

**Annex III: Tigrigna version of information sheet, consent form and questionnaire**

**መእተዊ ሓደ: ሐበሬታ ብዛዕባ ቃለ-መሕተት**

**ክፍሊ1: ሓበሬታ ብትግሪኛ**

ሰላም ኣነ _________________________ልተብሃልኩ ናይ ንጉስ ኣለሙ ዝተብሃለ ናይ ኣዲስ ኣባባ ዩኒቨርስቲ ካልኣይ ድግሪ ተመራቒ መመረቒ ጽሑፍ ሓደሓደ ነገር ካባኹም ክሓትት ስለዝደለኹ ፍቓድኩም ብትሕትና ይሓትት፡፡

**አከያይዳ፡-**

ነዚ ቃለ መሕትት ዝመረጽናኩም ብኣጋጣሚ እምበር ኮነ እልካ ከምዘይኮነ ፡ብተወሳኺ እዉን ብፍቓድኩም ምዃኑ ክኒሕብረኩም ንፎቱ፡ ኣብ ዝኾነ ግዜ ድማ ነቲ ስምምዕ ክተፍርስዎ ትኽእልዎ እኹም፡

በዚ መሰረት እንተተስማዕምዖም ሓደሓደ ሕቶታት ክንሓቶም እና፡እቶም ሕቶታት ናይ ግሊ ህወትን ናይ ሕማሞም ኩነታት ዝምልከት ይኸዉን. ካብዚ ብተወሳኺ እስካብ ሓሙሽተ ሚሊሊትር ደም ቀድሕና ክንወስድ እና. እዚ ደም እዚ ድማ ናይ ሽኮር ሕማም ዘለኩም ሰባት ናይ ደም ዋሕዲ ምህላዉን ዘይምህላዉን ንምርግጋፅ ንምርመራ ዝውዕል ይኸውን. ነዚ ስራሕ ዚ ንምስላጥ ብጠቕላላ ካብ 20 እስካብ 30 ደቓይቕ የሓትት፡፡

**ድሕኒነት ብዝምልከት፡-**

እዚ ፅንዓት እዚ ኣብ ነካይደሉ ግዜ ምንም ዓይነት ጉድኣት ከምዘይብሉ ከምኡ ድማ ዝኾነ ዘስግእ ዓይነት ነገር እንተተሰምዕዎም ኣበዝኾነ ግዜ ምቁራፅ ከም ዝኽእሉ ክነረጋግፀልኩም ንፎቱ፡፡

**ጥቕሚ ብዚተመልክተ፡-**

ኣብዚ ፅንዓት እዙይ ምንም ዓይነት ክፍሊት ኣይኽፈልን.ነገር ግን እዚ ፅንዓት ምስተጠናቐቐ ንኹሎም ናይዚ ሕማም ተጣቃዕቲ ሓዉሱ ንብዙሓት ተገልገልቲ ብዛዕባ ዋሕዲ ደም ዕዙዝ ሓበሬታ ን ጥቕሚን ከምዘበርኪት ንኣምን፡፡

**ኣተሓሕዛ ሚስጥር ብዝምልከት፡-**

ኣብዚ እትህብዎ ሓበሬተታ ይኹን ካልእ ብምስጥር ከምዚትሓዚን ስምኩም ኮነ ካልእ መለለዪ ኣበወረቐት ከምዘይሰፍር ብዝኾነ መንገዲ ድማ ሚስጥርኩም ከምዘይነውፅእ ንገልፀልኩም ኒፎቱ. ተወሳኺ ሓበሬታ እንተደልኹም እዉን ን ዋና ተመራማሪ እዚ ሰራሕ ዝኾኑ ኣይተ ንጉስ ኣለሙ በዚ 0924342979 ስልኪ እዚ ምድዋል ይክኣል፡፡

ፍቓዶም/ደን እንተኾይኑ ናይ ስምምዕ ፍርማ ኣብዚ ወረቐት ይፈርሙ::

**1. እሺ.............. (ቐፅል)**  **2. ኣይከዉንን................ (ኣ**ቋ**ርፅ )**

**ክፍሊ ክሊተ፡ናይ ስምምዕ ዉዕሊ ዚገሊፅ ፍርማ**

ብመሰረት ኣብ ላዕሊ ዝተውሃበኒ ሓበሬታ ናይዚ ፅንዓት ዕላማ፤ጥቕሚን፤ጉድኣትን ብምርዳእ፣ንዝኾነ ዝቐርበለይ ሕቶ ንምምላስ፤ ከምኡ ውን ኣድላዪ ንምርመራ ዝኸውን ደም ንምሃብን ከምዝተስማዕማዕኹ ብ ፍርማይ የረጋግፅ፡፡

ሽምን ፍርማን ተማራማሪ ኣካል**______________________, _________**

**ናይ መላሲ ፍርማ ____________________________**

ናብ ቃለ መሕተት ክቕፅል ፍቓድኩም/ደን ድዩ?

**1.እወ.......................... (ቃለ-መሕተት ይቕፅል)**

**2.ኣይፋሉን................... (የቐንየለይ፣ኣ**ቋ**ርፅ)**

**ናይ ቃለ-መሕተት ውፅኢት ዝምልከት:-**

1.ዘተማልአ___________________________

2.ፍርቂ ዝተማልአ_____________________

ቃለመጠይቕ ዝጅምረሉ ስዓት______________________

ቃለ መጠይቕ ዝውደአሉ ስዓት______________________

1ይክፋል መጠየቒ ቂጥዒ: ብዛዕባ ግላዊ ሓበረታ ዝምልከት

| **ዕድመ ብ ዓመት** |  | **ሃይማኖት** | 1.ኦርቶዶክስ |
| --- | --- | --- | --- |
|  |  |  | 2.ሞስልም |
| **ጠፀታ** | 1.ተባዕታይ |  | 3.ካቶልክ |
|  | 2.ኣነስታይ |  | 4.ካልእ___ |
| **ዝነብርሉ ቦታ** | 1. ከተማ | **ብሄር** | 1.ትግራይ |
|  | 1. ገጠር |  | 2.ኣፋር |
| **ናይ ትምህርቲ ኩነታት** | 1.ብስሩዕ ዜተምሃረ |  | 3.ኣምሃራ |
|  | 2.ቀዳማይ ደረጃ ዘጠናቐቐ |  | 1. ካልእ___ |
|  | 1. ካልኣይ ደረጃ ዘጠናቐቐ | **ናይ ስራሕ ኩነታት** | 1.ናይ መንግስቲ |
|  | 1. ኮለጅ ዘጠናቐቐ |  | 2.ናይ ግሊ |
|  | 1. ማስተርስ ዲግሪ |  | 3.ተምሃራይ |
| **ናይ ሓዳር ኩነታት** | 1. ዘይተመርዓወ |  | 1. ማዕልታዊ ሰራሕተኛ |
|  | 1. ዝተመርዓወ |  | 6.ነጋዳይ |
|  | 3.ዝተፋትሐ |  | 1. ሓለስታይ |
|  | 4.ዝተፈላለየ |  | 1. ካልእ_____ |
|  | 5.ሓዳር ዘይገበረ | **ኣታዊ ኩነታት** | 1.ላዕለዋይ |
|  |  |  | 2.ማእከላይ |
|  |  |  | 3.ታሕተዋይ |

**ክፍሊ 2: ክሊኒካል ኩነታት ዝምልከት ሓበሬታ**

| **ናይ ሕሙም ሓበሬታ** | **ምላሽ** | **ኩነታት ሕሙም** | **ምላሽ** |
| --- | --- | --- | --- |
| ዓይነት ሽኮር | 1. ታይፕ 1 | መድሓኒት ብ አግባቡ ይወስዳ/ዱ/ | 1. እወ |
|  | 1. ታይፕ2 |  | 1. አይወሰድን |
|  |  | ሕማም ሽኮር ካበ ዝህልዎመ ክንደይ ገይረን/ሮም? | 1. 5 ዓመት |
| ዓይነት ዝወስድዎ መድሓኒት | 1. ሌንተ |  | 1. 5-10 ዓመት |
|  | 1. ሜትፎርሚን |  | 1. >10 ዓመት |
| ካልእ ዝፍለጥ ሕዱር ሕማም አለዎም/ወን ዶ | 1. እወ | ሕዱር ናይ ዓይኒ ሕማም ኣለወን/ዎም? | 1. እወ |
|  | 1. የብለይን |  | 1. የብለይን |
| ሽጋራ የትክኻኹ | 1.እወ |  |  |
|  | 2.ኣይትኪክን | ኤች አይ ቪ ከነታት | 1. ፖዘቲቭ |
| ፀቕጢ ደም ኣለዎም/ወን ዶ | 1.እወ |  | 1. ነጋቲቭ |
|  | 2.ኣይብለይን | ሄማቶክሪት መጠን ብ ሚግዴሊ |  |
| ሄሞግሎብን መጠን ብ ሚግዴሊ |  |  |  |
|  |  | መጠን ክብደት ብ ኪግ | ________ |
| መጠን ድፍእት ደም ብ ሚሜ |  | ቁመት ብ ሴሜ | _______ |
| አብ 3 ወርሒ ውሽጢ ፀረ ሓሳኹ ወስደን /ዶም? | 1. እወ 2. አይወሰድኩን | አልኮል ይሰተዩ/ያ? | 1. እወ 2. አይሰትን |
