# Supplementary material for: The magnitude of anemia and associated factors among adult diabetic patients in Tertiary Teaching Hospital, Northern Ethiopia, 2019, cross-sectional study
Source: PLoS One. 2020 Nov 10;15(11):e0240678. doi: 10.1371/journal.pone.0240678 (PMC7671748; doi:10.1371/journal.pone.0240678)
Supplement: Attachment — Submitted filename: Response to Reviewers.docx [file pone.0240678.s003.docx]

**Response to Reviewers**

| Reviewer1’s comment | response |
| --- | --- |
| 1. The authors declare that they don’t have specific funding source. However, they claim in the acknowledgement section that they have funding source from Addis Ababa University and Adigrat. This conflict of depiction need to be resolved. | We have changed that the funding was from Addis Ababa University (internal organization) and we corrected the cover letter also according this. But the funder had no role in study design, data collection, analysis and decision to publish or preparation of the manuscript. |
| 2. Abstract: The authors ascertained that “anemia is higher in diabetes patients attending Ayder   Comprehensive Specialized Hospital” before the study conducted which is a wrong premise. How the author explains their claim about the existence of a high incidence of anemia among DM patients before the study conducted Vs the need to conduct the study to determine the magnitude?  In general, in the abstract part the problem statement and justification should be provided unambiguously. | Thank you very much dear. We accept the comments and corrected accordingly. |
| 1. In the discussion part, the authors should support “free radicals formation due to DM” with reference(s). | Here, reference number 32 is used to serve for that whole paragraph and to make it clear and specific I add that reference for that sentence specifically. |
| **Reviewr2’s comments** | **Response** |
| Abstract - Under background section: The authors stated that anemia is higher in diabetes patients ... and its magnitude is less emphasized. This can't be relevant reason to investigate this problem. If the magnitude is known and higher as stated by the authors, what is needed just intervention not research - The objective is presented in the abstract and not shown in the introduction. Even here the formulated objective for magnitude but the title and result the authors presented include risk factors for anemia. Therefore, either the objective should be revised or the title and result should be revised - Under result part of abstract the presented AOR for some of the variable is very wide. Does the author understand the meaning of wide CI and take care while concluding this study? - Under conclusion section: It is stated that "... anemia is higher"; higher than which group? - It is also stated "...Human Immune Deficiency Virus infection... . as associted with anemia." Bu not indicated in the result section | Here in the abstract we are writing after analyzing the result, and we found there is high magnitude of anemia among diabetic patients. You were right if this was abstract of proposal. The magnitude is just known after this research. This is not a plan, this is just a result.  In the introduction part, we didn’t include the objective and we include it now after your comments.  And the objective is amended and made in line with the title according your comments.  Some of the AORs in the result part are wide and we tried repeatedly but that was a result, and we can’t manipulate results, we accept it as it is.  In the conclusion we are not comparing with other group, this is a cross section study. There are not groups to compare with. There was an editorial problem “higher” but now corrected to be high.  Had it been a case control study or else, we could have a comparison group but now, it is a cross sectional study, and we don’t have any group.  HIV is included now in the result part. |
| The aim of the study was not shown | Now the aim is included at the end of the back ground. |
| The number of DM patients and care given for the patients must be outlined so that the reader can easily understand the sampling technique, and so on | The comment is accepted and the total number of patients is included in the latest revised manuscript, which is 2400. |
| How did the researcher assessed whether the medication is anti-helminthic or not? | The data collectors checked the chart of the patients, and they knew that it was anti-helminthic from their diagnosis by the physicians.it was documented in their chart. |
| Please show the interval and sampling procedure | Simple Systematic sampling was used and every 9^th^ patient was included in the study. |
| Please describe when and where the data was collected from the patients - In page four paragraph two, it is presented that "And all the anthropometric and BP measurements were measured twice." Then which one was used in the analysis? | The data was collected in February and march 2019. And it was collected in Ayder Comprehensive Specialized Hospital, Northern Ethiopia.  all the anthropometric and BP measurements were measured twice and their average was taken in to final analysis. |
| What is the outcome and independent variables. | The outcome variable is anemia, and all the other variables are the independent ones, including the sociodemographic variables. |
| in Table 1: How did you measure income? What is high, medium or low mean?? How did the author categorize it? | I used local classification of the Central statistics of the region, which is less than 1500.~~00~~ ETB lower,1500-3000.~~00~~ ETB,middle income and those who get above 3000.~~00~~ per month were higher income. |
| Very long and less informative table. Example: Table 2: Bivariate analysis of variables for anemia among diabetes mellitus patients in ACSH, 2018/19, (n=262); | We accepted the comment and removed the table. |
| The author is has no objective or missed the aim. The title of the table says anemia but what is presented seems mean Hb. | The measurement of Hb is used to see the anemia status of the patients. |
| I don't see the advantage of this table. If needed the crude and adjusted result can be presented using one table | We already removed it. |
| The author compared two different non-comparable setting. Ex prevalence anemia in Ethiopia versus England. Look at paragraph 3 | We are trying to show the difference between the developed and developing nations. |
| Statistically non-significant variables were discussed as if they were. Example look at the following paragraph:"type-2 diabetic patients  have 1.7 times (AOR, 1.67, 95%CI, 0.81-3.3, P=0.17) high chance of anemia than type-1 diabetic patients" | We accept and we removed it.  Thank you! |
| Or some of the justifications that were literature based were not cited. Look at paragraph 3. Ex "In this study prevalence of anemia was found to be higher in females (11.5% vs. 6.4%), and this is due to the biological factors like menstrual loses in those who were in their reproductive age. In addition the higher levels of androgen in males may contribute to the lower prevalence of anemia among males." | Here we didn’t cite the references because this is just our possible justification, not just the reason of others work. And this is explained by scientific knowledge of the authors. |
| the author is expected to present the strength and limitation of the study | Ok. Thank you. We add it at the end of the discussion part. |
| Must made revised in line with update of document | It is revised based on the updated document. |
| Please follow the guideline and correct the referencing | Ok. We follow as per the guide line as much as possible.  Thank you! |
| Please amend your Response to Reviewers letter to include a point by point response to each of the points made by the Editor and / or Reviewers | We include all points in this response letter. |
| At this time we ask that you please upload your minimal data set as a Supporting Information file | We said we can give a data upon a reasonable request; this is not the reasonable request to upload our data. |
| Please amend the title either on the online submission form or in your manuscript so that they are identical. | We made it as per your comment. |
| Please ensure that you refer to Figure 2 and 3 in your text as, if accepted, production will need this reference to link the reader to the figure. | We amend it and we are referring the respective figures. And their labels are below the figures. |
| Please include a legend for figure 5 and 6. | We already excluded the figures from the manuscript. |
| Please upload a copy of Figure 5 and 6 which you refer to in your text on page 10. Or if the figure is no longer to be included as part of the submission please remove all reference to it within the text. | All the figures are removed because we revise it and found to be less significant to include the figures. The idea of the figures is conceptualized and rephrased in words. |
| Yes - all data are fully available without restriction" "All relevant data are within the manuscript and its Supporting Information files." | The data are fully available; the data is already given to you as per your request. |
