# Supplementary material for: The magnitude of anemia and associated factors among adult diabetic patients in Tertiary Teaching Hospital, Northern Ethiopia, 2019, cross-sectional study
Source: PLoS One. 2020 Nov 10;15(11):e0240678. doi: 10.1371/journal.pone.0240678 (PMC7671748; doi:10.1371/journal.pone.0240678)
Supplement: Attachment — Submitted filename: 00Response to Reviewers8.docx [file pone.0240678.s004.docx]

**Response to Reviewers**

| Reviewer1’s comment | response |
| --- | --- |
| 1. Please amend the title either on the online submission form or in your manuscript so that they are identical. | We accept the comment, and amended accordingly. Now the title in the manuscript is identical with the title online.  Thank you. |
| 2. Please amend your list of authors on the manuscript to ensure that each author is linked to an affiliation.   We note that you have included affiliation numbers 1-5, ⃰, &, and ¶ however only affiliations 1-5 have authors liked to them. Please amend affiliations ⃰, &, and ¶ to link an author to it or remove if added in error. | Yes. We also accept the comments and amended as of your recommendation. Now the numbers 1-5 and the symbols ⃰ ,¶, and & are linked to the affiliation of each author.  Thank you. |
| 3. Please ensure that you refer to Table2 in your text as, if accepted, production will need this reference to the link the reader to the Table. | Yes. The text is referring to table 2. And now made an amendment and rearranging of the text. We added a statement on the top to show that the text is referring to Table2. |
