# Supplementary material for: The magnitude of anemia and associated factors among adult diabetic patients in Tertiary Teaching Hospital, Northern Ethiopia, 2019, cross-sectional study
Source: PLoS One. 2020 Nov 10;15(11):e0240678. doi: 10.1371/journal.pone.0240678 (PMC7671748; doi:10.1371/journal.pone.0240678)
Supplement: Attachment — Submitted filename: Reviewer comment-plos one.docx [file pone.0240678.s002.docx]

**Title:** Magnitude of Anemia and Associated Factors among Adult Diabetes Patients in

Tertiary Teaching Hospital, Northern Ethiopia, Cross Sectional Study

**Reviewer General Comments:** The authors work may have significant contributions to understand the level of anemia cases among DM patients in Ethiopia set-up. Given the limited existence of similar researches in Ethiopia, this research outcome will have importance for devising or revising health policy in the country, specifically in Ayder Hospital. Considering the strong experimental design and the aforementioned reasons, I recommend the paper to be consider for publication after a minor revision.

On the other hand, the study could have been more strong were the authors recruited a control group (non-DM subjects).

**Specific comments:**

1. The authors declare that they don’t have specific funding source. However, they claim in the acknowledgement section that they have funding source from Addis Ababa University and Adigrat. This conflict of depiction need to be resolved.
2. Abstract: The authors ascertained that “anemia is higher in diabetes patients attending Ayder

Comprehensive Specialized Hospital” before the study conducted which is a wrong premise. How the author explain their claim about the existence of a high incidence of anemia among DM patients before the study conducted Vs the need to conduct the study to determine the magnitude?

In general, in the abstract part the problem statement and justification should be provided unambiguously.

3. In the discussion part, the authors should support “free radicals formation due to DM” with reference(s).
